# Assessing Nutritional Diversity of Cropping Systems in African Villages

**DOI:** 10.1371/journal.pone.0021235

**Published:** 2011-06-16

**Authors:** Roseline Remans, Dan F. B. Flynn, Fabrice DeClerck, Willy Diru, Jessica Fanzo, Kaitlyn Gaynor, Isabel Lambrecht, Joseph Mudiope, Patrick K. Mutuo, Phelire Nkhoma, David Siriri, Clare Sullivan, Cheryl A. Palm

**Affiliations:** 1 The Earth Institute at Columbia University, New York, New York, United States of America; 2 Leuven Sustainable Earth Research Center at Katholieke Universiteit Leuven, Leuven, Belgium; 3 Institute of Evolutionary Biology and Environmental Studies, University of Zurich, Zurich, Switzerland; 4 Division of Research and Development, CATIE, Turrialba, Costa Rica; 5 World Agroforestry Centre, The Sauri Millennium Villages Cluster, Kisumu, Kenya; 6 Bioversity International, Rome, Italy; 7 Department of Earth and Environmental Sciences at Katholieke Universiteit Leuven, Leuven, Belgium; 8 The United Nations Development Program, The Ruhiira Millennium Villages Cluster, Mbarara, Uganda; 9 The Millennium Development Goals Centre for East and Southern Africa, Nairobi, Kenya; 10 The United Nations Development Program, The Mwandama Millennium Villages Cluster, Zomba, Malawi; Tulane University, United States of America

## Abstract

**Background:**

In Sub-Saharan Africa, 40% of children under five years in age are chronically undernourished. As new investments and attention galvanize action on African agriculture to reduce hunger, there is an urgent need for metrics that monitor agricultural progress beyond calories produced per capita and address nutritional diversity essential for human health. In this study we demonstrate how an ecological tool, functional diversity (FD), has potential to address this need and provide new insights on nutritional diversity of cropping systems in rural Africa.

**Methods and Findings:**

Data on edible plant species diversity, food security and diet diversity were collected for 170 farms in three rural settings in Sub-Saharan Africa. Nutritional FD metrics were calculated based on farm species composition and species nutritional composition. Iron and vitamin A deficiency were determined from blood samples of 90 adult women. Nutritional FD metrics summarized the diversity of nutrients provided by the farm and showed variability between farms and villages. Regression of nutritional FD against species richness and expected FD enabled identification of key species that add nutrient diversity to the system and assessed the degree of redundancy for nutrient traits. Nutritional FD analysis demonstrated that depending on the original composition of species on farm or village, adding or removing individual species can have radically different outcomes for nutritional diversity. While correlations between nutritional FD, food and nutrition indicators were not significant at household level, associations between these variables were observed at village level.

**Conclusion:**

This study provides novel metrics to address nutritional diversity in farming systems and examples of how these metrics can help guide agricultural interventions towards adequate nutrient diversity. New hypotheses on the link between agro-diversity, food security and human nutrition are generated and strategies for future research are suggested calling for integration of agriculture, ecology, nutrition, and socio-economics.

## Introduction

While great strides in reducing hunger through increases in agricultural productivity have been made worldwide, more than 900 million people are undernourished [1], over 2 billion people are afflicted by one or more micronutrient deficiencies [2] and over 1 billion adults are overweight [3]. In addition to producing sufficient calories, a major, often overlooked challenge in agriculture and food systems is to provide an adequate diversity of nutrients necessary for a healthy life. A human diet requires at least 51 nutrients in adequate amounts consistently [4]. It has been argued that changes in agricultural production systems from diversified cropping systems towards ecologically more simple cereal based systems have contributed to poor diet diversity, micronutrient deficiencies and resulting malnutrition in the developed as well as developing world [4]–[7]. Success of agricultural systems has historically been evaluated primarily on metrics of crop yields, economic output and cost-benefit ratios [8]. Yet, these metrics do not reflect the diversity of nutrients provided by the system that is critical for human health. In this study we take a step to demonstrate how ecological tools can play a role in addressing nutritional diversity as an overlooked ecosystem service of agricultural systems.

In nutritional sciences, several methods have been developed that look beyond the single nutrient or food item to capture the broader picture of diet diversity [9]–[13]. Count measures are frequently applied to assess diet diversity, where the number of consumed food items and food groups is recorded [9]. Diet quality indices have also been developed that take into account consumption pattern and nutritional composition of food items [10], [11]. Numerous studies have shown that nutritional quality of the diet improves as a higher diversity of food items or food groups is consumed [14]–[18] and increased diet diversity has been associated with positive health outcomes such as lower rates of stunting, mortality, and incidence of cancer [18]–[24].

Approaches to quantifying diet diversity in nutrition research have direct analogs to approaches to quantifying biological diversity in ecology. Counting total number of food items or food groups is analogous to counting species richness and functional group richness. In ecology, there is increasing interest in quantitative measures of functional diversity, which take advantage of the wealth of information available on species' traits, particularly for plants, to overcome some of the drawbacks or lack of sensitivity of the simpler measures of diversity [25]. Among these quantitative approaches is the functional diversity metric FD [26]. FD is a metric that reflects the trait distinctiveness of a community and the degree of complementarity in traits of species within a community.

Here we introduce a novel nutritional functional diversity metric (nutritional FD). The nutritional FD metric is based on plant species composition on farm and the nutritional composition of these plants for 17 nutrients that are key in human diets and for which reliable plant composition data are available (Table 1). We use this FD metric to summarize and compare the diversity of nutrients provided by farms in three sites in Sub Saharan Africa (SSA). The nutritional FD value increases when a species with a unique combination of nutrients is added to a community, and decreases when such a species is lost. Changes in the presence or absence of species with identical nutritional composition do not change the value of FD, however such redundancy provides a buffer, in case other species are lost from the system. For example, changing climate conditions could prevent some plant species from being successfully cultivated, so having several species with similar nutritional composition means that such a shift in crop species composition would not necessarily impact the overall nutritional diversity at the farm or community level. The nutritional FD metric thus reflects the diversity of nutrients provided by the farm and the complementarity in nutrients among species on a farm or community.

**Table 1:** Nutrients and nutrient groups taken into account for calculation of FD metrics.

Macronutrients	Minerals	Vitamins
Protein	Calcium (Ca)	Vitamin A
Carbohydrates	Iron (Fe)	Vitamin C
Dietary fibre	Potassium (K)	Tiamin
Fat	Magnesium (Mg)	Riboflavin
	Manganese (Mn)	Folate
	Zinc (Zn)	Niacin
	Sulfur (S)	

From the 51 required nutrients for human diets, 17 nutrients that are key for human diets and for which reliable plant composition data were available in the literature were selected. Because plants are not a proven source for Vitamin B12 and Vitamin D, these were not included.

The three sites examined here, Mwandama in Malawi, Sauri in Kenya, and Ruhiira in Uganda, are part of the Millennium Villages Project (MVP), where food insecurity and under-nutrition rates are high [27]–[29]. A principal goal of the MVP is to improve food security and nutrition through a set of interventions recommended by the United Nations Millennium Project Hunger Task Force [30]. The sites represent distinct but representative agro-ecosystems of SSA (Table 2), with maize (Mwandama, Sauri) or banana (Ruhiira) as the staple crop. Subsistence farming is the main livelihood strategy for over 75% of the households in these sites [27]–[29]. On average 50% of food consumed in the household comes from own production and 75% of food consumed in the village comes from production within the village (Table 2).

**Table 2:** Site characteristics.

	Malawi, Mwandama	Kenya, Sauri	Uganda, Ruhiira
Farming system and Agro-ecological zone	Cereal root-crops mixedSubhumid Tropical	Maize mixedSubhumid tropical	Banana-based Highland perenial
Major crops	Maize	Maize, Beans	Banana
Rainfall pattern and annual average (mm)	Unimodal1139	Bimodal1800	Bimodal1050
Altitude (m above sea level)	900–1200	1400	1350–1850
Average area cropped per household (ha)	1.0	0.6	1.9
Average % of food consumed by the *household* that comes from own production (calculated in $ values)	46%	35%	69%
Average % of food consumed in the *village* that comes from production in the village (calculated in $ values)	70%	75%	82%
Dominant soils and fertility conditions	Rhodustalfs, loamy to clayey	Rhodic Hapludox, clayey	Rhodic Hapludox and Acrisols, sandy clay loam
Soil pH	5.25 (±0.60)	5.74 (±0.37)	5.45 (±0.85)
Soil Effective Cation Exchange Capacity (ECEC)	5.74 (±2.34)	7.03 (±1.96)	13.63 (±4.34)
Soil % Nitrogen (N)	0.079 (±0.026)	0.121 (±0.031)	0.260 (±0.066)
Soil % Carbon (C)	1.098 (±0.415)	1.461 (±0.332)	3.078 (±0.742)
Soil C/N ratio	13.91 (±2.18)	12.39 (±2.20)	11.96 (±1.27)

Soil values represent average scores ± standard deviation based on 60 samples [29], [65].

In this study we explore how nutritional FD metrics can provide insights in nutrient diversity of farming systems and can have potential to guide agricultural management. Data on plant species diversity, food security and diet diversity were collected for plots and home gardens of 170 farms in Mwandama, Sauri and Ruhiira and iron and vitamin A deficiency was determined from blood samples for 30 adult women per village. Four nutritional FD metrics were calculated: FD_total_ describing diversity for all 17 nutrients of Table 1, FD_macronutrients_ for the four macronutrients, FD_minerals_ for the seven minerals and FD_vitamins_ for the six vitamins. Differences between farms and villages for species richness, nutritional FD, household food and health indicators were analyzed as well as relationships between these different indicators.

## Methods

### Research sites

The Mwandama village cluster is located in southern Zomba district of Malawi and covers an approximate population of 35,000 people. The region once characterized by native Miombo woodlands is now intensively cultivated. Smallholders grow mainly maize, pigeon peas, cassava, and groundnuts, while commercial estates produce tobacco and maize. Livestock management is practiced on a small scale and is restricted to chicken and goats.

The Sauri cluster is located in the Kenyan highlands in the western Nyanza Province and has a farm community of 63,500 people. The main occupations are subsistence farming, consisting primarily of maize, sorghum and cassava, and animal husbandry, including goats, chickens, and cattle.

The Ruhiira cluster is situated in the Isingiro District in the hilly, dissected terrain of southwest Uganda and has a population of approximately 43,056 people. The agricultural system is predominantly a mixed system with livestock and cultivation of annual and perennial crops. The main crop is banana, which covers approximately 30% of the total cropland.

Further site characteristics are outlined in Table 2 [27]–[29].

### Sample selection and data collection

A random sample of 50 to 60 farms per site was selected based on demographic and geographic MVP data for 300 previously randomly selected households per cluster. For Ruhiira and Mwandama data for 60 farms were collected during June–September of 2009. For Sauri data for 50 farms were collected during November of 2009. A full explanation of the study procedures, purpose, risks and benefits were explained to participants during the informed consent process. The study received ethical approval from the Institutional Review Board at Columbia University.

### Documentation of species diversity

For each of the 170 farms, all plots, including home gardens, cultivated by the household, were sampled to document all crop, plant and tree species, with different species and varieties according to local definitions. Plant species were confirmed with the help of local botany studies [31]–[37]. In addition, it was noted if these plants were edible and consumed by the household. Only plants that were edible and consumed in the village were considered for this study.

### Nutritional trait data of plants

A database of plant nutritional composition data was developed based on existing studies and databases (Supporting information Box S1). When different parts of certain plants were consumed, both parts were listed and taken into account in further calculations. The nutritional composition data were standardized and weighted by converting values to the percentage of the Dietary Reference Intake (DRI) [38] for the specific nutrient provided by 100 g of the consumable product. So, for each nutrient, % of DRI provided by 100 g of that plant species were the values used to calculate the FD scores. Seventeen nutrients were selected based on data availability and the essential role they play in human diets (Table 1).

### Calculation of diversity metrics

**Figure 1:**
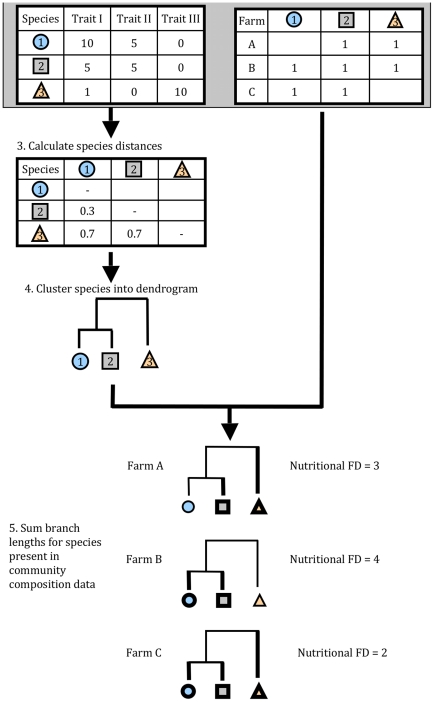
Schematic model of how to assess nutritional functional diversity. Two data sets are required: a species by trait matrix (1), and a farm or site by species matrix (2). From the species×trait matrix, the multivariate distances between crop species are calculated (3), where distance is a function of distinctness in nutrient composition and content. The distances between species are used to cluster species into a dendrogram (4). Based on the crop species present in a given farm, the branch lengths of the dendrogram are summed (5). Example Farms A and C illustrate how nutritional functional diversity can differ even when species richness is identical, depending on the nutritional distinctiveness of the crop species present.

Species richness was defined by the number of identified and previously described edible species per farm. Petchey and Gaston's FD [26] was used as a measure of nutritional functional diversity, with 17 nutrients from 77 crops (Figure 1). Functional diversity metrics begin with two data matrices: 1) a species by trait matrix, and 2) a farm or site by species matrix [39]. In the method we used here, the species × trait matrix is used to calculate the multivariate distances between crop species, where distance between a pair of species determined by the distinctness in nutrient composition and content. Then the distances between species are used to cluster species into a dendrogram, which reduces the dimensionality of the diversity metric calculation. Finally, based on the crop species present in a given farm, the branch lengths of the dendrogram are summed, to give the FD value (Figure 1).

In the crop nutritional data set we use here, the species×trait matrix is composed by the % of DRI for a specific nutrient. The community composition matrix contains the presence of absence of each crop species for each of the 170 farms. We calculated nutritional FD in four ways: using all 17 nutrients, using just the four macronutrients, using the six vitamins, and using the seven minerals (Table 1), resulting in four respective FD metrics: FD_total_, FD_macronutrients_, FD_minerals_ and FD_vitamins_. Results were scaled by the maximum values to range from 0–100 for each FD metric separately. The dendrogram for FD_total_ generated in this analysis is available as supporting information (Figure S1).

### Functional redundancy and observed versus expected FD

**Figure 2:**
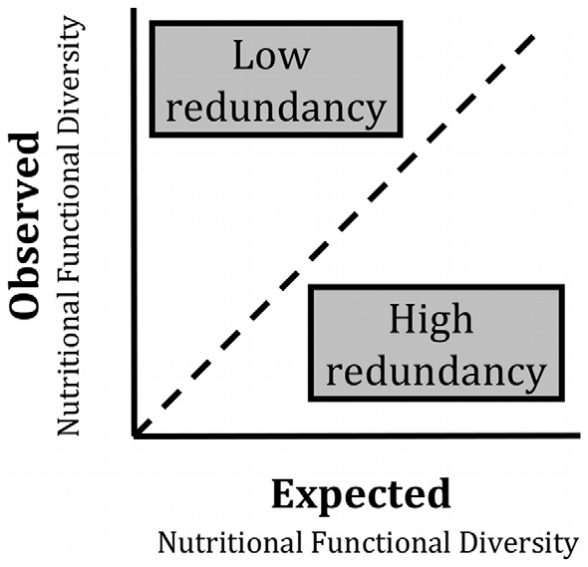
Schematic model to assess degree of redundancy by modeling observed versus expected functional diversity for a given species richness. If a set of communities has a large range of species richness, but shows little variation in functional diversity, then the species pool in that set of communities has high functional redundancy. In contrast, a set of communities with low functional redundancy may exhibit large changes in functional diversity with only small changes in species richness.

We assessed the degree of functional redundancy by simulations that model observed versus expected functional diversity for a given species richness (Figure 2) [40]. To calculate “expected FD” scores, we used a simulation approach to create a null distribution of FD values for the observed number of species. Holding species richness constant for each of the 170 households, we randomly selected species without replacement from the species pool (the total number of species in the study) to calculate a null FD value for each household. We repeated this 5,000 times to produce a distribution of null values and tested whether the observed FD for each household was significantly higher or lower than the null FD distribution, at α = 0.05 [40]. For this study, “expected FD” is thus the mean of the functional diversity calculated from many possible species combinations for a particular number of species.

This approach allows us to determine if changes in FD across households simply reflect species richness, or if species composition and trait diversity vary in other ways e.g., with village or other factors. If a set of communities has a large range of species richness, but shows little variation in functional diversity, then the species pool in that set of communities has high functional redundancy (Figure 2). That is to say, many species share similar traits and the loss of a few species has little impact on functional diversity. In contrast, a set of communities with low functional redundancy may exhibit large changes in functional diversity with only small changes in species richness (Figure 2) [40].

### Household food indicators

Recommendations of the Food and Nutrition Technical Assistance (FANTA) project were used to develop questionnaires for the months of inadequate household food provisioning (MIHFP, range 0–12; adapted from months of adequate household food provisioning [41], household food insecurity access scale (HFIAS, range 0–21) [42] and household diet diversity score (HDDS, range 0–15) [9] based on a 24 hour recall for consumption of 15 food groups: cereals; vitamin A rich vegetables and tubers; white tubers, roots and plantains; green leafy vegetables; other vegetables; vitamin A rich fruits; other fruits; legumes and nuts; oils and fat; meat; fish; eggs; milk; sweets; spices and tea [9]. The surveys were first pre-tested and adapted to local conditions and language.

### Iron and Vitamin A deficiency

Individual serum samples were collected from 30 women between the ages of 13 to 49 per site (90 in total) to determine iron and vitamin A deficiency.

Iron was measured by a colorimetric assay using the Hitachi 917 analyzer (Roche Diagnostics, Indianapolis, IN). Under acidic conditions, iron is liberated from transferrin. Ascorbate reduces the Fe^3+^ ions to Fe^2+^ ions, which then react with FerroZine reagent to form a colored complex. The color intensity is directly proportional to the iron concentration in the sample and is measured photometrically. Iron at the concentration of 46; 93 and 138 ug/dL has a day-to-day variability of 1.8%; 1.1% and 0.6%, respectively. Iron deficiency was defined as a level less than 15 ng/mL [43].

The levels of vitamin A were measured by high performance liquid chromatography (Shimadzu Corporation, Kyoto Japan). Vitamin A is de-proteinized from the serum/plasma sample using ethanol and extracted with hexane. The extract is dried, re-dissolved with ethanol and injected into the chromatograph. Retinyl acetate is used as the internal standard. This assay is standardized using calibrators from the National Institute of Standards and Technology. The minimum required volume for this assay is 150 microliters. Vitamin A deficiency was defined as a level <20 micrograms/dL [43].

All calculations, as well as general linear models and analysis of variance, were done in the statistical programming environment R (2.11.0, www.r-project.org).

## Results

### Species diversity

Across the 170 farms of the three sites, a total of 77 edible, previously described plant species were identified (Supporting information Table S1). Twenty-seven of these 77 species were common among all three sites. The average number of edible species per farm differs significantly between villages, ranging from 11 in Mwandama to 18 in Ruhiira (Table 3). Farm species richness was found to be independent from farm landholding size (r^2^ = −0.0017, p = 0.366), also when corrected for village. The five most commonly grown crops across all three sites are bananas (on 93% of the farms), maize (91%), beans (75%), cassava (75%), and mango (69%). Examples of unique species for one of the sites include several green leafy vegetables such as *Corchorus olitorius* (apoth) and *Crotalaria brevidens* (mito) for Sauri in Kenya; tamarillo or tree tomato (*Solanum betaceum*) and some spices e.g., ginger and cardamom, for Ruhiira in Uganda; certain fruits such as peaches, figs and pomegranates for Mwandama in Malawi (Supporting information Table S1).

**Table 3:** Indicator outcomes per site.

		Malawi, Mwandama	Kenya, Sauri	Uganda, Ruhiira	p-value
Edible plant diversity in village	Edible species richness of village (number of unique species for that site)	42 (11)	49 (11)	55 (13)	
Edible plant diversity per household farm	Edible species richness	11.15±3.66	15.22±4.29	18.25±4.82	<0.001
	Nutritional FD_all_ [0–100]	49.25±17.96	64.56±16.32	68.44±15.82	<0.001
	Nutritional FD_macronutrients_ [0–100]	46.73±9.75	52.7±13.15	72.23±14.54	<0.001
	Nutritional FD_minerals_ [0–100]	32.21±10.56	52.52±16.14	70.88±16.2	<0.001
	Nutritional FD_vitamins_ [0–100]	41.97±24.48	46.91±17.92	45.78±18.08	0.41
Household food indicators	HHDDS	7.57±2.58	8.22±2.05	9.2±3.18	<0.001
	HHFIS	11.65±5.80	7.62±5.01	10.27±4.96	<0.001
	MIHFS	4.37±2.27	2.56±2.18	3.97±1.67	<0.001
Nutritional health indicators	Vit A deficiency women	0.00%	3.30%	6.70%	0.563
	Fe deficiency women	23.30%	6.70%	6.70%	<0.001

Values represent total number for indicators at the village level and average scores for indicators at the household ( = farm) or individual level ± standard deviation. P-values are shown for ANOVA test of village effect on farm/household/individual level indicators. HHDDS: Household Diet Diversity Score; FIS: Household Food Insecurity Score, MIHFS: Months of Inadequate Household Food Supply.

### Nutritional FD and relationship with species richness

Four nutritional FD metrics (FD_total_, FD_macronutrients_, FD_minerals_ and FD_vitamins_) were calculated for each of the 170 farms (Table 3). This approach allows us to investigate the nutritional diversity across all nutrients and within each of the major nutrient groups. For three out of these four FD metrics, average values for farms differs significantly between the sites (p<0.001) (Table 3), with equivalent values only for FD_vitamins_ (p = 0.41). Similar to species richness, all FD metrics were found to be independent from farm landholding size (p>0.1).

**Figure 3:**
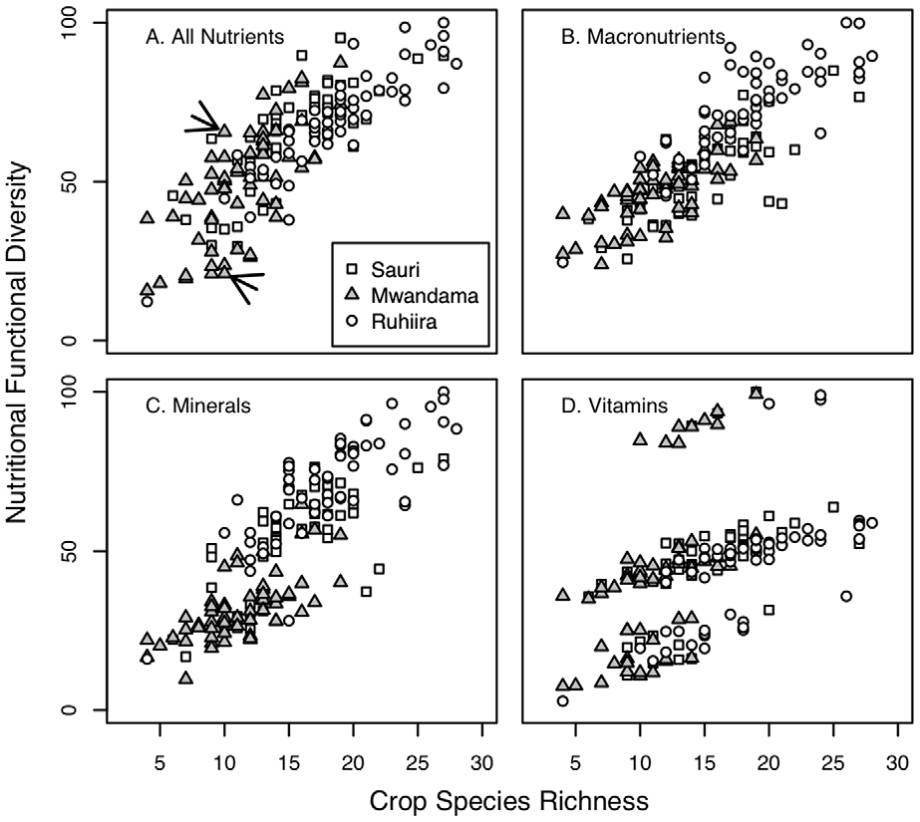
Nutritional functional diversity values are plotted against species richness for 170 household farms. A: Nutritional FD = FD_total_, summarizing functional diversity for all 17 nutrients listed in table 1; B: Nutritional FD = FD_macronutrients_ for the four macronutrients; C: Nutritional FD = FD_minerals_ for the seven minerals; D: Nutritional FD = FD_vitamins_ for the six vitamins (table 1). Farms in Mwandama are shown as triangles, farms in Sauri as squares, and farms in Ruhiira as circles.

Figure 3 plots FD values against species richness for each of the 170 farms. Regression of FD_total_ (Figure 3A) against species richness reveals several patterns. First is a strong positive correlation (p<0.001; r^2^ = 0.68) between FD_total_ and species richness, independent of village. Thus, as the number of edible species increases, the diversity of nutrients that farm provides also increases. Second, at a level of around 25 species per farm, the relationship between FD_total_ and species richness starts leveling off, meaning that additional species to a farm, with around 25 or more species, increases nutritional diversity very little. Third, although species richness and FD_total_ are correlated, farms with the same number of species can have very different nutritional FD scores. For example, two farms in Mwandama (indicated by arrows on Figure 3A) both with 10 species show an FD_total_ of 23 and 64, respectively. The difference in FD is linked to a few differences in species nutritional traits. Both of these example farms grow maize, cassava, beans, banana, papaya, pigeon pea and mango. In addition, the farm with the higher FD score grows pumpkin, mulberry, and groundnut, while the farm with lower FD score has avocado, peaches and black jack (in Malawi, black jack leaves are consumed). Trait analysis shows that pumpkin (including pumpkin leaves, fruits and seeds which are all eaten) adds diversity to the system by its relatively high nutritional content in vitamin A, Zn, and S-containing amino acids (methionine and cysteine) compared to other species; mulberry by its levels of vitamin B complexes (thiamin, riboflavin) and groundnut by its nutritional content for fat, Mn, and S. The black jack, avocado and peaches found in the lower FD farm add less nutritional diversity to the system than pumpkin, mulberry, and groundnut since they do not contain the vitamin B or S complexes, and thus are less complementary to the other plants in the system for their nutritional content. This example shows how different crop species compositions can result in very disparate nutritional FD even with identical numbers of crops planted in a field.

When considering the FD values based on the nutrient subgroups, i.e. macronutrients, minerals and vitamins, the pattern of the relationship between species richness and FD differs among subgroups (Figure 3B, C and D). While FD_macronutrients_ increases nearly linearly with increasing species richness, FD_vitamins_ shows abrupt changes and is highly dependent on the presence of few species. For example, addition of mulberry or guava species strongly increases the FD_vitamins_ value of the farm because of their unique high values for vitamin B complexes and vitamin C, respectively. This uniqueness attributed to a few key species results in a stepwise pattern of different FD_vitamins_ levels instead of a gradual increase with number of species and indicates high species sensitivity (see also below). For FD_minerals_, the group of farms in Mwandama differs significantly from the Ruhiira and Sauri farms, by lower FD_minerals_ values and a lower slope in the FD_minerals_ - species richness relationship (p<0.001). This suggests that the species on the Mwandama farms are not contributing as much mineral diversity to the system than species in the Kenya or Uganda village (see also below).

### Functional redundancy

**Figure 4:**
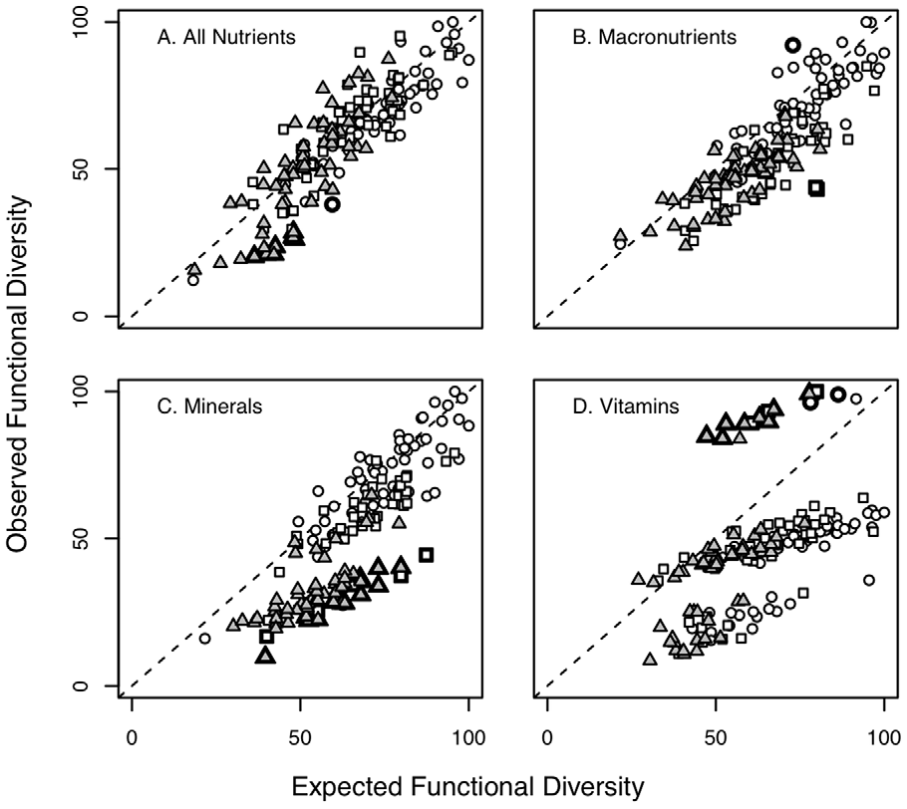
Observed values for nutritional diversity are plotted against simulated expected nutritional FD values for 170 household farms. Farms that have observed FD values significantly different from expected FD values are marked in bold. Farms in Mwandama are shown as triangles, farms in Sauri as squares, and farms in Ruhiira as circles.

A crucial component of FD is functional redundancy [44], which reflects the degree of overlap in the traits of species in a community. We assessed the degree of functional redundancy by simulations that model observed versus expected functional diversity for the each of the 170 farms and the four nutritional FD metrics (Figure 4). When observed FD is higher than expected, it indicates low functional redundancy, or that species are more distinct from one another than expected by chance (Figure 2) [40].

Figure 4 illustrates that functional redundancy patterns differ among nutrient groups. For FD_total_ and FD_macronutrients_ no strong redundancy patterns are observed (Figure 4A, B). For FD_minerals_, a group of farms (in bold in Figure 3C) with an observed FD significantly lower (at α = 0.05) than the expected FD was identified, meaning there is high functional redundancy, with several species having similar nutrient traits. Most of these farms are of the Mwandama site, and in contrast to other farms, they are entirely lacking a set of species identified as most influential for mineral diversity including *Sesamum calycinum* (onyulo) which is particularly rich in Fe, *Eleusine coracana* (finger millet) with high Ca and Mn levels, *Glycine max* (soybean) rich in Fe, Mg and Mn, *Helianthus anuus* (sunflower) which seeds have high levels of Zn, Mg and S and *Solanum nigrum* (black nightshade) rich in Fe and Mn.

In contrast to the pattern of high redundancy for FD_minerals_, for FD_vitamins_ a group of farms (in bold in Figure 4D) can be identified with significantly higher observed FD than expected FD, meaning there is low functional redundancy on those farms as only a few species provide certain combinations of vitamins (Figure 4D). What these farms have in common is that they all contain the species *Morus alba* (mulberry). As mentioned above, mulberry, especially the leaves, contain vitamins B complex and C, in higher levels than most other plants. Addition or loss of mulberry as one of the few species in the community providing vitamin B complex, can increase or reduce FD_vitamins_ significantly.

### Linking to food and health indicators

In addition to agro-biodiversity data, data on household food indicators including a household food insecurity access scale (HFIAS), number of months of inadequate household food provisioning (MIHFP) and household diet diversity scores (HHDDS) were obtained for each of the 170 farms (Table 3). Significant differences between villages for these indicators reflect different levels in food security and diet diversity, with lower food security and diet diversity in Mwandama as compared to Ruhiira and Sauri (Table 3). Average village data indicate that low species richness and FD scores at the village level are paired with low diet diversity, high food insecurity and number of months of inadequate food provision of the village community (Table 3).

Analysis of correlations between these household food indicators and farm species richness and nutritional FD metrics indicate that for each of the food indicators, correlation coefficients are slightly higher for FD metrics than for species richness. But none of these correlations are significant and significance does not change when corrected for village and/or land size (data not shown).

The patterns for iron and vitamin A deficiencies at the village level (Table 3) are similar to patterns for FD_minerals_ and FD_vitamins_ respectively: while Mwandama shows significantly higher rates of Fe deficiency than Ruhiira and Sauri, average FD_minerals_ of Mwandama farms is significantly lower compared to FD_minerals_ in Ruhiira and Sauri. No significant differences between sites are found for Vitamin A deficiency and similarly, FD_vitamins_ is the only FD metric for which the three sites score equally.

## Discussion

Sub-Saharan Africa faces pressing challenges, with 40% of children chronically undernourished or stunted [45]. As new investments and attention galvanize much-needed action on African agriculture, a vigorous debate is required to ensure that agricultural progress is evaluated based on metrics that go beyond economic cost/benefit ratios and calories per person and that can also address the complexity of nutritional diversity required for human health. In this study, we demonstrate how an ecological concept, the FD metric, has potential to summarize nutritional diversity of cropping systems and thereby provide new insights on provisioning ecosystem services across farms and villages in Sub-Sahara Africa.

The strengths of the study lie in the development of a systems approach that is able to consider the large variety of species available in the system together with their nutritional composition and in the step it takes towards integrating agriculture, nutrition and ecology studies [46], [47]. By applying the FD metric on nutritional diversity, it was possible to identify variability in nutritional diversity across farms and villages (e.g., low diversity for minerals in the Mwandama cluster compared to Sauri and Ruhiira) as well as to identify species that are critical for ensuring the provisioning of certain nutrients (e.g., mulberry for vitamin B complexes). The results also emphasize that the species nutritional composition and redundancy available in the system determine if introduction or removal of certain species will have critical impacts on the nutritional diversity of the community (e.g., addition of species to farms with around 20 species does not cause much change to FD_total_, high species sensitivity for FD_vitamins_, high redundancy for FD_minerals_).

While in the past, food-based interventions in developing countries have focused mostly on a single nutrient [5], the approach described in this study can help guide agricultural interventions towards diversity of nutrients and/or towards nutrient redundancy or resilience of the system. In particular, this work provides means to identify potential crops, varieties or groups of plants that add nutritional value (diversity or redundancy) and can be introduced, promoted, or conserved taking into account the functional diversity of species already available in the system. The single nutrient approach of the past, varying from various recommendations for high-protein diets [48] and later for high-carbohydrate diets [49], [50], to more recent efforts directed at the elimination of micronutrient deficiencies, was in part linked to a lack of knowledge in earlier years about the interactions among nutrients in human physiology and metabolism [5]. The roles of micronutrients in health and well-being and the synergies in their physiologic functions are now being increasingly recognized, supporting the notion that nutrient deficiencies rarely occur in isolation and calling for dietary diversification [5], [51]–[54]. These advances in nutritional sciences also create a demand for applying a more holistic approach to the nutritional diversity of agricultural systems as described here.

This study is, however, limited and offers room for improvement on several fronts. First, no data were collected on the quantities produced or on species evenness. Cropping area or yield data would further strengthen the study by allowing calculation of an abundance-weighted FD metric, several of which have been developed in community ecology [55], [56]. While this is planned as a next step in future work, presence/ absence-based FD metrics are valuable as predictors of ecosystem functioning [57]. The nutritional FD metric of this study gives thereby valuable insights on the diversity of nutrients provided by the cropping system, particularly on the complementarity and redundance of species in the system and on the potential of species to contribute nutritional traits to the existing composition of species (on farm or in the village).

Second, the nutritional composition data and FD metric calculations were based on available species level data. It is known that a large diversity in nutritional composition exists among different varieties of species as well as among different environments in which plants are cultivated [58]–[60]. For example, certain varieties of *Phaseolus vulgaris L.* (common bean) are significantly higher in iron and zinc than other *P. vulgaris* varieties [4], [61], and addition of zinc fertilizer to the soil can further increase the concentration of trace elements in edible parts [62]. Also, the FD calculation used here does not take into account the level of neutriceuticals and phytochemicals, that play a beneficial role for human health, nor the level of anti-nutritional factors (e.g., phytate, oxalate, tannins) that reduce the bioavailability of certain nutrients (e.g., Ca, Fe, proteins). Efforts to acquire more data at the species and subspecies level on nutritional composition across different environments, will allow fine-tuning the proposed FD metrics. In addition, including livestock diversity and number will provide a more complete picture of the nutritional diversity available on farm.

No significant correlations at the farm level were found between nutritional FD of crops grown and household food consumption indicators. This might be partly due to limitations of the proposed FD metrics or the relatively simple household food indicators used in this study, but also to the complex pathway between agricultural production and food consumption [63], [64]. While most households in the studied villages are considered subsistence farmers, farm households are not closed systems. Food consumption and expenditure data (Table 2) show that the average proportion of food consumed coming from own production is around 50%. Also, a significant correlation was found between the number and value of food items bought and sold on local markets and the household food indicators at each of the three sites (FIS, HHDDS, MHIFS) [65]. These findings emphasize the importance of local markets and support the notion that these farm households are not closed systems. The most appropriate scale to link nutritional FD metrics to food consumption and nutrition indicators, would be the “foodshed”, defined as the geographic area that supplies a population center with food [66]. Village level data show that for example for Ruhiira 82% of food consumed is derived from production within the village (Table 2). This indicates that in the case of these villages, the foodshed, largely overlaps with the village. It is therefore interesting to note that certain associations between nutritional FD and food and nutrition indicators were observed at the village level: the correspondence in patterns between FD_minerals_ and Fe deficiency, FD_vitamins_ and vitamin A deficiency and FD_total_ and diet diversity, food insecurity and number of months of insufficient food supply. These findings generate new hypotheses on the link between nutritional diversity of the farming system and nutrition outcomes at the village or in particular the foodshed level such as: Can the high rate of Fe deficiency among adult women in Mwandama be due partly to a lack of species that contribute more significantly to mineral diversity, particularly those high in Fe? Also, does the high crop species richness in Sauri and Ruhiira play a role in their relatively lower level of food insecurity?

In addition, the study triggers new questions as to what are the determinants or filters of nutritional diversity on farms, villages and agro-ecological zones. For example, it is clear that mineral diversity of species in the Mwandama village is lower than in Sauri and Ruhiira, and even when species richness increases, FD_minerals_ in Mwandama remains relatively low. Several potential barriers for growing species that add more to FD_minerals_ can be hypothesized and could be categorized under ecological (e.g. climate, soil, altitude, water availability), dispersal (large distance to origin of seeds) or anthropogenic determinants (e.g. cultural preference, limited economic access to seeds, lack of knowledge). In this context, it is interesting to note that soil fertility measures in the Mwandama village (Table 2) show very low values for effective cation exchange capacity (ECEC) and percentage of total nitrogen (N), two factors that are critical for soil fertility. It can be hypothesized that the soil conditions in Mwandama restrict successful cultivation of crops to only those adapted to lower soil fertility conditions or it might be that farmers' preference for certain crops or soil management strategy has impacted soil fertility over time.

**Figure 5:**
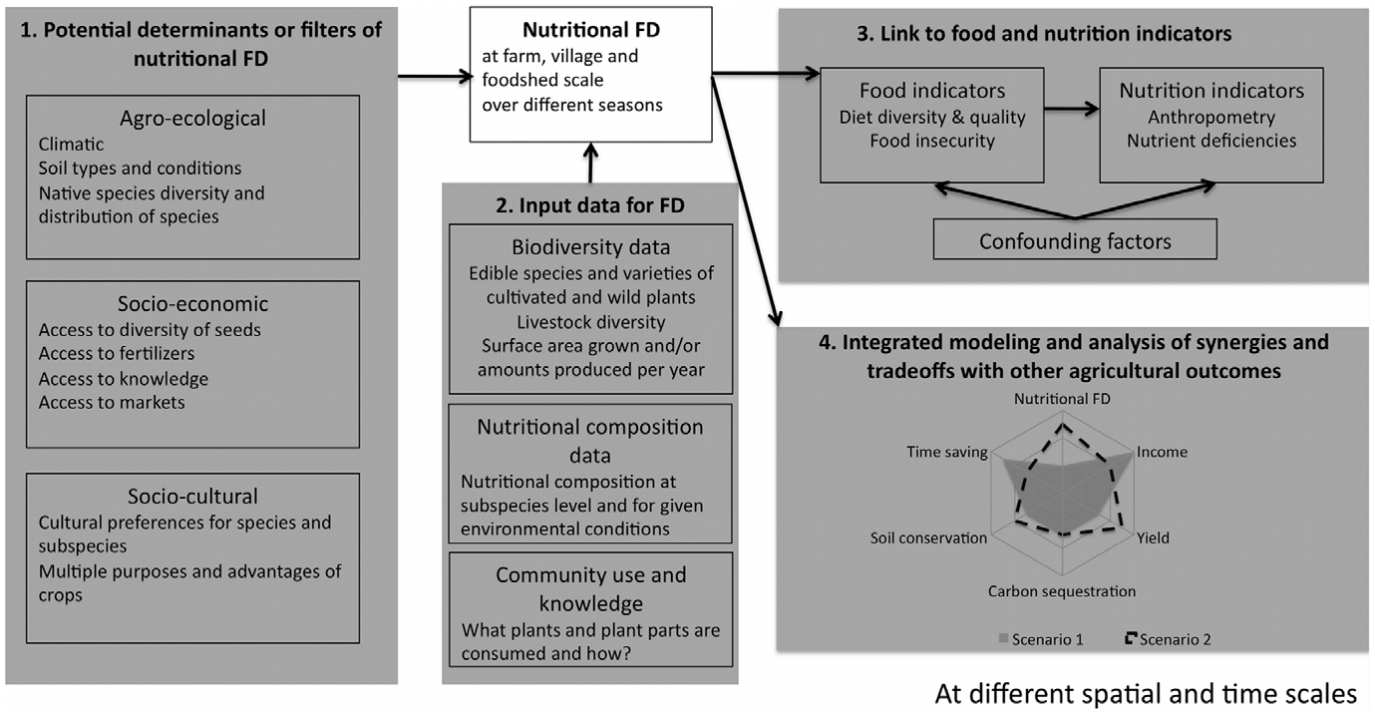
Suggested strategy for future research on nutritional functional diversity. The overall objective of the strategy is to guide agricultural and landscape interventions towards more balanced nutritional outcomes. Three major fronts for research are suggested: study of potential determinants and barriers of nutritional FD and identify the ones that can be controlled (1); collection of new and mobilization of existing data that enable a more comprehensive calculation of nutritional FD and this at a landscape and village level (2); establishing linkages with consumption and human health outcomes of agricultural systems through integrated datasets that include health and socio-economics (3); and integrated modeling and analysis of potential synergies and tradeoffs between nutritional diversity and other outcomes from agriculture (4).

Based on the findings and new questions raised, a strategy for future research is outlined in Figure 5, with an overall objective to guide more balanced nutritional outcomes from agricultural systems. The strategy emphasizes four major fronts for expanding the research presented here: 1) study on potential determinants and barriers of nutritional FD in different settings; 2) collection of new and mobilization of existing data that enable a more comprehensive calculation of nutritional FD across different villages; 3) establishing linkages between nutritional diversity of farming systems and consumption and human health outcomes, particularly at the foodshed scale [66], [67] and 4) integrated modeling and analysis of potential synergies and tradeoffs between nutritional diversity and other outcomes from agriculture e.g. income generation, risk reduction, greenhouse gas emissions, water quality, labor intensity, and social well-being. Such modeling can be done at different scales (farm, village, country, region, global) and across agro-ecological zones to identify how complementary different agro-ecosystems are for providing the necessary nutritional diversity.

In conclusion, this study delivers novel work on addressing nutritional diversity of agricultural systems. We show that applying the ecological functional diversity metric on nutritional traits of plants in agricultural systems gives insights on the diversity of nutrients provided by cropping systems. Application of this metric can help guiding management decisions towards increased nutrient diversity for a given number of species, as well as towards increased redundancy or buffer of species for a specific set of nutrients.

In addition, new hypotheses on the link between agro-biodiversity and nutrition are generated and a cross-disciplinary research framework is suggested. Nutritional FD is thereby a tool that bridges agriculture, human nutrition and ecology studies and offers an entry point for integration of other scientific disciplines (economics, anthropology, human health, landscape ecology) [47], [68], [69]. Assessing the multiple outcomes of agricultural systems across agro-ecological zones is critical for making progress towards more sustainable and nutritious food systems [70].

## Supporting Information

Box S1 **List of main sources used to establish nutritional composition database used in this study.**
(DOC)

Figure S1 **Dendogram for nutritional FD_total_ taking into account the 17 nutrients listed in** **Table 1** **.** Species are abbreviated as first three letters of genus plus first three letters of species. Full species names are outlined in Table S3.
(TIF)

Table S1 **List of edible, characterized plant species that were identified at the study sites.**
(XLS)
