# Supplementary material for: Assessing Nutritional Diversity of Cropping Systems in African Villages
Source: PLoS One. 2011 Jun 16;6(6):e21235. doi: 10.1371/journal.pone.0021235 (PMC3116903; doi:10.1371/journal.pone.0021235)
Supplement: Box S1 — List of main sources used to establish nutritional composition database used in this study. (DOC) [file pone.0021235.s001.doc]

**Major Databases**

**Food Composition Table for Use in Africa**

FAO, US Dept of Health, 1968

<http://www.fao.org/docrep/003/X6877E/X6877E00.htm>

**Tanzania Food Composition Table**

Muhimbili University of Health and Allied Sciences, Tanzania Food and Nutrition Centre, and Harvard School of Public Health, Nov. 2008

<https://apps.sph.harvard.edu/publisher/upload/nutritionsource/files/tanzania-food-composition-tables.pdf>

**DTU Food**

The official Danish Food Composition Database, 2009

<http://www.foodcomp.dk/v7/fcdb_default.asp>

**NUTRIBASE**

The Titi Tudorancea Bulletin

<http://www.tititudorancea.com/z/nutrition.htm>

**NewCROP Crop Index**

Purdue University Center for New Crops and Plant Products, 2008

<http://www.hort.purdue.edu/newcrop/Indices/index_ab.html>

**Traditional Food Plants of Kenya** (book)

P.M. Maundu, G.W. Ngugi, C.H.S. Kabuye

National Museums of Kenya, 1999

**Handbook of Nutrition and Diet** (book)

B.B. Desai.

CRC Press, 2000

Available on Google Books: <http://books.google.com/books?id=NrCkt1-UWikC&pg=PP1&dq=Handbook+of+Nutrition+and+Diet,+BB+Desai&ei=7p5xS8j-JoaszASFvOyUDg&cd=1#v=onepage&q=&f=false>

**Traditional Food Plant Foods of Canadian Indigenous Peoples: Nutrition, Botany, and Use**

H.V. Kuhnlein and N.J. Turner

Taylor & Francis, 1982

Available on Google Books: <http://books.google.com/books?id=fPDErXqH8YYC&dq=Traditional+plant+foods+of+Canadian+indigenous+peoples,+Kuhnlein+%26+Turner&source=gbs_navlinks_s>

**Scientific Publications**

**Multiple Species**

Freiberger, C.E.; Vanderjagt, D.J.; Pastuszyn, A.; Glew, R.S.; Mounkaila, G.; Millson, M.; and Glew, R.H. 1998. Nutrient content of the edible leaves of seven wild plants from Niger. *Plant Foods for Human Nutrition* 53: 57-69.

🡪 Includes information on the following species: *Ximenia americana, Amaranthus viridus, Corchorus tridens, Hibiscus sabdarifa, Maerua crassifolia, Moringa oleifera*, and *Leptadenia hastate*

Odhav, B.; Beekrum, S.; Akula, U.S.; and Baijnath, H. 2007. Preliminary assessment of nutritional value of traditional leafy vegetables in KwaZulu-Natal, South Africa. *Journal of Food Composition and Analysis* 20: 430-436.

🡪 Includes information on the following species: *Amaranthus dubius, Amaranthus hybridus, Amaranthus spinosus, Asystasia gangetica, Bidens pilosa, Centella asiatica, Ceratotheca triloba, Chenopodium album, Cleome monophylla, Cucumis metuliferus, Emex australis, Galinsoga parviflora, Justicia flava, Momordica balsamina, Oxygonum sinuatum, Physalis viscosa, Portulaca oleracea, Senna occidentalis, Solanum nodiflorum* and *Wahlenbergia undulata.*

Akindahunsi, A.A. and Salawu, S.O. 2005. Phytochemical screening and nutrient-antinutrient composition of selected tropical green leafy vegetables. *African Journal of Biotechnology* 4(6): 497-501.

🡪 Includes information on: *Basella alba, Hibiscus esculentus, Crassocephalum* crepidioides, *Occimum graticimum, Vernonia amygdalina, Solanum macrocarpon, Structium sparejanophora, Celocia argentea, Talinum triangulare, Corchorous olitorius, Piper guineese, Amaranthus caudatus, Manihot utilisima, Xanthosoma mafaffa*

Ojiako, O.A. and Igwe, C.U. 2007. Nutritional and anti-nutritional compositions of *Cleome rutidosperma, Lagenaria siceraria,* and *Cucurbita maxima* seeds from Nigeria. *Journal of Medicinal Food* 10(4): 735-739.

🡪 Includes information on *Cleome rutidosperma, Lagenaria siceraria,* and *Cucurbita maxima*

**Single Species**

***Amaranthus dubius***

de Arellano, M.L.; Albarracin, G.; Arce, S.L.; and Mucciarelli, S.L. 2001. Valor de una fuente alimenticia no convencional. *Revista Internacional de Botanica Experimental* 245-249.

***Launaea cornuta***

Ndossi, G.D. and Sreeramulu, N. 1991. Chemical studies on the nutritional value of *Launaea cornuta* – a wild leafy vegetable. *Journal of Food Science and Technology* 28: 183-184.

***Physalis peruviana***

Ramadan, M.F. and Morsel, J.T. 2003. Oil goldenberry (*Physalis peruviana* L.) *Journal of Agricultural and Food Chemistry* 51: 969-974.

***Solanum betaceum***

Vasco, C.; Avila, J.; Ruales, J.; Svanberg, U.; and Kamal-Eldin, A. 2009. Physical and chemical characteristics of golden-yellow and purple-red varieties of tamarillo fruit (*Solanum betaceum* Cav.) *International Journal of Food Sciences and Nutrition* 60(S7): 278-288.

***Solanum nigrum***

Akubugwo, I.E.; Obasi, A.N.; and Ginika, S.C. 2007. Nutritional Potential of the Leaves and Seeds of Black Nightshade – *Solanum nigrum* L. Var *virginicum* from Afikpo-Nigeria. *Pakistan Journal of Nutrition* 6(4): 323-326.
