# Supplementary figures and images for: Assessing Nutritional Diversity of Cropping Systems in African Villages
Source: PLoS One. 2011 Jun 16;6(6):e21235. doi: 10.1371/journal.pone.0021235 (PMC3116903; doi:10.1371/journal.pone.0021235)

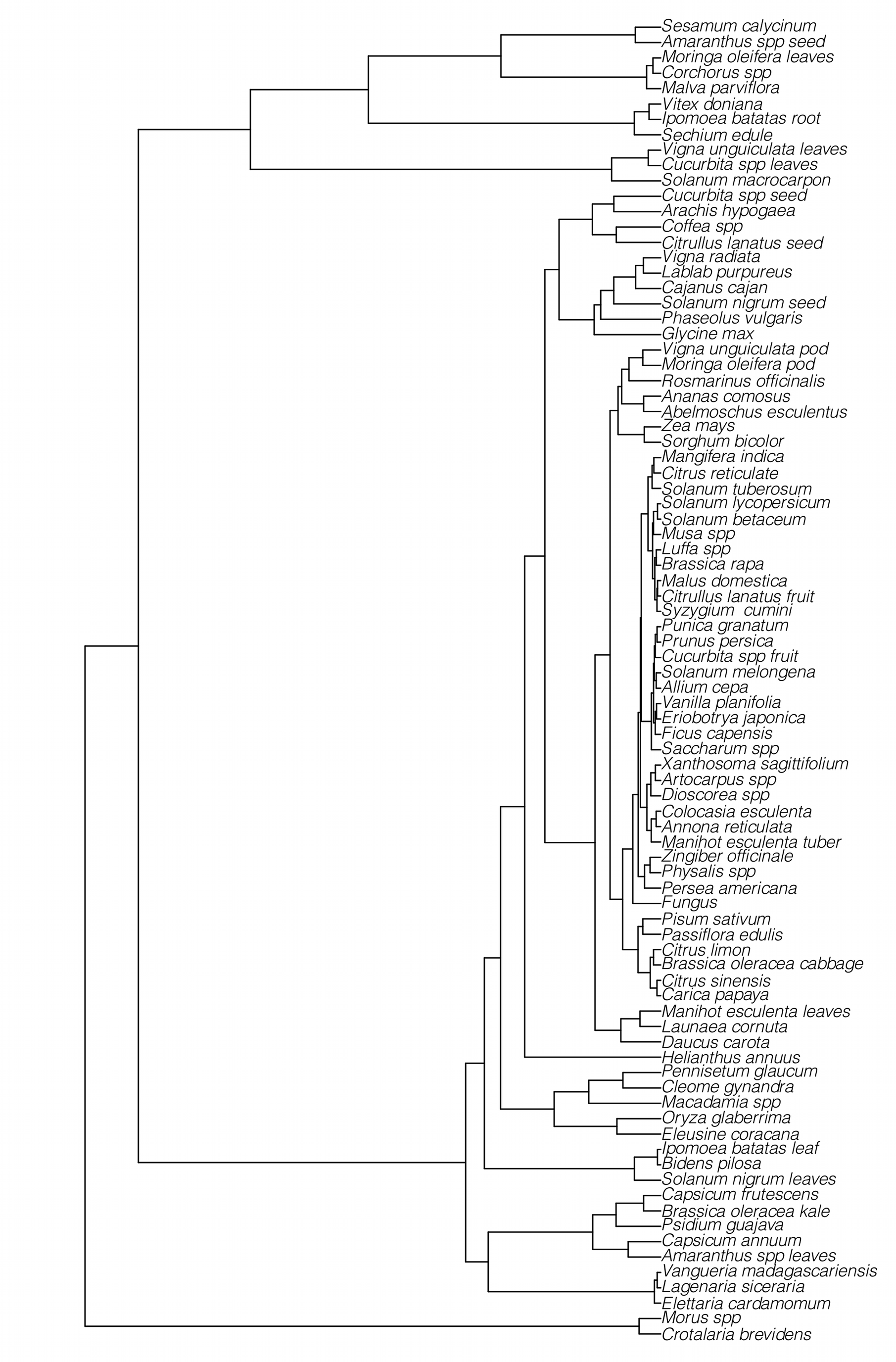

Supplement: Figure S1 — Dendogram for nutritional FDtotal taking into account the 17 nutrients listed in Table 1 . Species are abbreviated as first three letters of genus plus first three letters of species. Full species names are outlined in Table S3. (TIF) [file pone.0021235.s002.tif]
